# Art therapy in eating disorders. A systematic review of literature

**DOI:** 10.1192/j.eurpsy.2022.405

**Published:** 2022-09-01

**Authors:** F. Trably, P. Gorwood, L. Di Lodovico

**Affiliations:** GHU Paris Psychiatrie, Clinique Des Maladies Mentales Et De L’encéphale, Paris, France

**Keywords:** Psychotherapy, Eating Disorders, systematic review, art therapy

## Abstract

**Introduction:**

Art-therapy (encompassing plastic arts, music, theather and writing) is a promising and acceptable management strategy of eating disorders (ED). It has the potential to improve well-being and therapeutic alliance, targeting psychological dimensions of ED, and dealing with difficulties of expression and rationalization of patients. Nevertheless, the efficacy of this approach is difficult to evaluate because of the lack of studies in this area.

**Objectives:**

We sought to provide an overview on the efficacy of art therapy in the management of ED, by a systematic review of all controlled trials using art therapy on patients with ED.

**Methods:**

This systematic review included all controlled trials using art-therapy on a population of adolescent and adult patients with ED. The effect of art therapy on clinical indicators such as anthropometric variables, symptoms and dimensions of ED was evaluated.

**Results:**

Of the 1286 screened records, only four respected inclusion criteria. These four trials evaluated plastic art therapy, music therapy, writing therapy, and dance-movement therapy. A large number of bias and strong heterogeneity of inclusion criteria, techniques and variables prevented any attempt of quantitative synthesis. Music therapy appeared to have a significant effect on post-prandial anxiety, while dance-movement therapy showed an effect on body dissatisfaction.

**Conclusions:**

The generalizability of the results found is weakened by the high heterogeneity of trials. Replication studies and a rigorous methodologies are necessary for more reliable conclusions. Art therapy could help improving some specific dimensions of ED.

**Disclosure:**

No significant relationships.

